# Draft Genome Sequence of *Buttiauxella agrestis*, Isolated from Surface Water

**DOI:** 10.1128/genomeA.01060-14

**Published:** 2014-10-16

**Authors:** Narayanan Jothikumar, Amy Kahler, Nancy Strockbine, Lori Gladney, Vincent R. Hill

**Affiliations:** a National Center for Emerging and Zoonotic Infectious Diseases, Centers for Disease Control and Prevention, Atlanta, Georgia, USA; b IHRC, Inc., Atlanta, Georgia, USA

## Abstract

MI agar is routinely used for quantifying *Escherichia coli* in drinking water. A suspect *E. coli* colony isolated from a water sample was identified as *Buttiauxella agrestis*. The whole genome sequence of *B. agrestis* was determined to understand the genetic basis for its phenotypic resemblance to *E. coli* on MI agar.

## GENOME ANNOUNCEMENT

MI agar is a USEPA-approved agar for use with membrane filtration-based water quality analysis (1). *Escherichia coli* colonies appear blue due to dye cleavage by the β-D-glucuronidase enzyme. The agar also contains cefsulodin to suppress the activity of Gram-positive bacteria. MI agar has been reported to yield 5% false positive results (2,3). Reporting false positive *E. coli* results for drinking water creates a misleading characterization of risk from fecal contamination. Identification of such false positive isolates on MI agar can lead to formulations of enzyme-based chromogenic media with improved specificity for *E. coli* detection. In the present study we report the first whole genome sequence of *Buttiauxella agrestis* strain MCE isolated from a surface water sample, which exhibited the blue colony color that is characteristic of *E. coli* on MI agar. The isolate was identified as *B. agrestis* by *rpoB* gene sequencing (4). The bacterial isolate was grown overnight at 37°C in 10 mL Luria-Bertani broth to obtain 1 to 5× 10^8^ CFU/mL. The overnight culture was centrifuged and cells were pelleted and resuspended using 350 µL PBS. The culture was mixed with an equal amount of 2× UNEX nucleic acid extraction buffer (Microbiologics, MN) to extract DNA according to the manufacturer’s instructions and further purified using a polyvinylpolypyrrolidone (PVPP) spin column (Spin-IV-HRC columns, Zymo Research Corporation, Orange, CA).

Whole-genome sequencing of *B. agrestis* strain MCE was performed on Illumina platform (MR DNA Shallowater, TX). A library with an average insert size of 500 bp was prepared from 50 ng of DNA using a Nextera DNA sample preparation kit (Illumina Inc., San Diego, CA). The pooled library was loaded to a 600 Cycles v3 Reagent cartridge followed by paired-end sequencing with a read length of 2×300 bp on a MiSeq platform as per manufacturer’s instructions (Illumina). The draft whole genome sequence was obtained by *de novo* assembly of the trimmed paired-ends using DNAStar SeqMan NGen Software. The assembled genome contains 12 contigs (*N*_50_ of 1,606 Kb) and has a predicted genome size of 4.8 Mb. The genome sequence had an overall coverage of 450× and an average G+C content of 49.3%. Annotation was performed using the RAST (Rapid Annotation using Subsytems Technology) server (5). RAST predicted 4,385 coding sequences and 103 RNAs representing 567 subsystems based on functional roles. Resistance genes identified by RAST included antimicrobials [fluoroquinolones, β-lactams and fosfomycin (*fosA*)] and genes involved in resistance to heavy metals (arsenic, cobalt, zinc, cadmium, mercury, and copper). This sequence information will enable comparison of genomic differences between *E. coli* and *B. agrestis* to identify differences (e.g., metabolism of sugars, antibiotic resistance) that can be utilized to improve the specificity of *E. coli* detection methods.

### Nucleotide sequence accession numbers.

This whole-genome shotgun project has been deposited at DDBJ/EMBL/GenBank under the accession no. JPRU00000000. The version described in this paper is version JPRU01000000.
